# Feline Coronavirus and Alpha-Herpesvirus Infections: Innate Immune Response and Immune Escape Mechanisms

**DOI:** 10.3390/ani11123548

**Published:** 2021-12-14

**Authors:** Paolo Capozza, Annamaria Pratelli, Michele Camero, Gianvito Lanave, Grazia Greco, Francesco Pellegrini, Maria Tempesta

**Affiliations:** Department of Veterinary Medicine, University of Bari Aldo Moro, 70010 Valenzano, Italy; paolo.capozza@uniba.it (P.C.); annamaria.pratelli@uniba.it (A.P.); michele.camero@uniba.it (M.C.); gianvito.lanave@uniba.it (G.L.); grazia.greco@uniba.it (G.G.); francesco.pellegrini@uniba.it (F.P.)

**Keywords:** innate immunity, FCoV, FIPV, FeHV-1

## Abstract

**Simple Summary:**

Feline coronavirus (FCoV) and feline herpesvirus-1 (FeHV-1) can induce infections that are difficult to prevent and to treat due to the involvement of host genetic factors and immune mechanisms. These two viruses areimportant examples of viral immune evasion of the host’s innate immune response. The innate immune system provides an early form of host protection from infectious diseases without pre-exposure and plays an essential role in determining the outcome of viral infections. The mechanisms that the innate immune system utilizes to counteract infections are based on therecognition of a relatively limited set of molecular structures that are either products of microbes (virus, bacteria, fungi, parasites) or expressed by injured or dead host cells. This review provides a brief overview of the main mechanisms achieved by host’s innate immunity, focusing primarily on the immune escape mechanisms developed and carried out by FCoV and FeHV-1 during infection.

**Abstract:**

Over time, feline viruses have acquired elaborateopportunistic properties, making their infections particularly difficult to prevent and treat. Feline coronavirus (FCoV) and feline herpesvirus-1 (FeHV-1), due to the involvement of host genetic factors and immune mechanisms in the development of the disease and more severe forms, are important examples of immune evasion of the host’s innate immune response by feline viruses.It is widely accepted that the innate immune system, which providesan initial universal form of the mammalian host protection from infectious diseases without pre-exposure, plays an essential role in determining the outcome of viral infection.The main components of this immune systembranchare represented by the internal sensors of the host cells that are able to perceive the presence of viral component, including nucleic acids, to start and trigger the production of first type interferon and to activate the cytotoxicity by Natural Killercells, often exploited by viruses for immune evasion.In this brief review, we providea general overview of the principal tools of innate immunity, focusing on the immunologic escape implemented byFCoVand FeHV-1 duringinfection.

## 1. Introduction

Over time, feline viruses have gained opportunistic properties, rendering their infections particularly difficult to prevent and treat. The solitary lifestyle of ancestral felines has driven the evolutionof viruses with highly efficient viral transmission strategies and the induction of latent, chronic and/or asymptomatic infections, thus evading the host’s immune system and increasing the carrier population and viral dissemination [1,2,3,4].

Feline coronavirus (FCoV) and feline herpesvirus-1 (FeHV-1)—due to the involvement of a host genetic factors and immune mechanisms in the development of the disease and the more severe forms [5,6,7]—areimportant examples of immune evasion of the host innate immune response.

FCoV (family *Coronaviridae*, subfamily *Orthocoronavirinae*, genus *Alphacoronavirus*, subgenus *Tegacovirus*, species *Alphacoronavirus* 1) [8] is an enveloped, positive-sense, single-stranded RNA (ssRNA+), highly contagious virus [9], which causes, based on biotype, either asymptomatic infections and mild self-limiting enteritis (feline enteric coronavirus-FeCV) or more lethal feline infectious peritonitis (characterized by polyserositis, vasculitis and severe lymphopenia [1,10,11,12]. After oral-fecal transmission, FCoV determines an initial enteric infection and lately the virus can spread beyond the intestine, resulting in a monocyte-associated viremia, with or without the development of FIP [1,11,13]. Feline infectious peritonitis virus (FIPV) originates from a mutated FCoV [14], which after positive selection pressures undergoes a change intropism from the apical villi epithelium of the small intestine to monocytes/macrophages [13]. As FIP occurs, delaying by weeks or years, the viral and host factors combine to transform an enteritis in to an immune-mediated disease [5,13,14,15].

FCoV genome, approximately 29 kb, encodes 11 proteins with four structural proteins, namely spike (S), envelope (E), matrix (M), and nucleocapsid (N), and five non-structural proteins, namely replicase 1a and 1b polyproteins (which are enzymatically cleaved to produce 16 functional proteins involved in RNA synthesis), and accessory proteins 3a, 3b, 3c, 7a, and 7b [11,12,15,16,17]. ORF 7 proteins have been shown to be essential for efficient in vitro replication and for in vivo virulence [18]. The ORF3 proteins were found to have only supportive roles during FIPV infection of the target cell [19]. Spike, 3a-c and the membrane genes, and the 7a-b ORFs, are the main candidates for mutation, leading to FIP phenotype [11,16].

FeHV-1 (order *Herpesvirales*, family *Herpesviridae*, subfamily *Alphaherpesvirinae*, genus *Varicellovirus*, species *Felid alphaherpesvirus* 1) [8], an enveloped, double-stranded DNA (dsDNA) virus [20,21], is the agent of feline viral rhinotracheitis. FeHV-1 is considered to be one of the most important ocular and upper respiratory pathogens of domestic cats worldwide [22], resulting in severe morbidity and mortality, especially in kittens [23]. FeHV-1 infects and replicates in the upper respiratory tract of susceptible cats, leading to fever, sneezing, and nasal and ocular discharge [24,25]. The virus spreads rapidly through the oral, nasal, and ocular secretions of infected cats [25,26]. In 6- to 9-week-old cats, clinical signs associated with FeHV-1 infections include common neurological signs, fever, pneumonia, ocular lesions, and a high fatality rate [23,26], due to loss of passive immunity [27,28]. As with other herpesviruses, latency is established in trigeminal ganglia after the acute phase of infection. Stress or immunosuppression readily leads to virus reactivation, with the shedding of infectious virus and recrudescence of clinical signs [28].Vaccines implemented against FeHV-1 can reduce the severity of symptoms without preventing infection, and the onset of latency and reactivation [28]. Based on the alpha-herpesviruses literature and the homologous function covered by herpesviral genes within the Herpesviridae family, glycoprotein E (gE), glycoprotein C (gC), thymidine kinase (TK) and serine/threonine protein kinase (PK) have been identified as potential virulence factors [28].

In recent years, molecular assays and the metagenomic approach have allowed researchers to unearth several virulence-associated genes. While in FCoV genes, mutations occur during viral replication due to error-prone viral polymerase lacking proofreading ability [9,16], in FeHV-1 they are present in the genome, and their deletion results in a significant reduction of replication and cytopathic effect in feline respiratory epithelial cells [28].

It is widely accepted that the innate immune system, which provides an initial universal form of the mammalian host protection from infectious diseases without pre-exposure, plays an essential role in determining the outcome of viral infection [29]. Although several studies have been conducted in mouse models, the antiviral innate immune system of cats remains under-investigated, with several open questions regarding the initial responses to viral infection and the possibility of manipulating the host’sinnate immune mechanisms [30].

The aim of this article is to provide a brief overview of the main mechanisms carried out by innate immunity, focusing on the immunologic escape carried out by FCoV and FeHV-1 during viral replication.

## 2. Immune Response

The immune response always starts with the necessary recognition of the foreign microorganism. During evolution, organisms developed diversified systems for recognizing nucleic acid invaders [31]. This is witnessed by the presence of defense mechanisms against DNA viruses [31,32] in lower organisms (i.e., bacteria) and by the presence of an ever-increasing range of defense mechanisms against DNA and RNA viruses in higher organisms, in particular vertebrates [31,32,33,34]. The large presence of RNA viruses and their massive replication within cells, which could lead to cell death, have led cells to develop relatively sophisticated defense mechanisms. Framing viruses, the recognition systems, although also present in free soluble form, are mostly concentrated at the cellular level, some distributed on the cell surface, others present both at organelle level and free in the cytoplasm [33,34].

### 2.1. Pattern Recognition Receptors

An important component of the innate system is the pattern recognition receptors (PRRs) network, which is able to detect microbial structures conserved in entire classes of microorganisms, and their associated signaling pathways leading to interferon (IFN) and inflammatory gene expressions [31,33,35]. PRRs targets are common products of metabolic pathways unique to a particular class of microbes, such as lipopolysaccharide, in the case of Gram-negative bacteria [33]. As for viruses, which are obligatory intracellular pathogens, all viral molecular components are produced in the host cell. Consequently, their detection by PRRs occurs more often in the intracellular space and the main targets are viral nucleic acids [32,33]. There are several classes of viral sensing PRRs, including Toll-like receptors (TLRs), RIG-I-like receptors (RLRs), C type lection receptors (CLRs), inflammasomes and DNA sensor [31,33].

### 2.2. Toll-Like Receptors

TLRs are the best characterized members of the PRR family, recognizing conserved molecular patterns associated with a variety of microorganisms [33,36]. TLR recognition of microbial ligands results in the activation of several signaling pathways and, finally, transcription factors, which induce the expression of genes whose products are important for inflammatory and antiviral responses [33].

All TLRs are type I transmembrane proteins, with three structural elements, a hydrophobic ectodomain containing leucine-rich repeats for ligand binding, a transmembrane domain, and a Toll-interleukin-1 receptor resistance (TIR) domain in the cytoplasmic tails to activate intracellular signaling [33,36]. Ligand-induced TLR dimerization leads to TIR domains of the cytoplasmic tails of each protein close to one another. The enrollment of TIR domain-containing adaptor proteins, such as Myeloid Differentiation primary response 88 (MyD88), TIR-domain-containing adapter-inducing interferon-β(TRIF) and TNF receptor associated factors (TRAF), facilitate the recruitment and the activation of various protein kinases, leading to the activation of different transcription factors [36].

There are 10 functional TLRs in humans and 12 in mice [31,33]. Although the genes encoding TLRs have been best studied in mice and humans, they have also been identified in other vertebrates, including cats [34,37]. Feline TLR genes 1 to 9 have been isolated and found variously distributed and expressed in several tissues, with different functions [37].

TLRs can be expressed on the cell surface, with the ability to detect bacterial, fungal, and protozoa products, and in endosomes, with the potential to detect nucleic acid [31,32,33,34]. TLRs 1, 2, 4, 5, and 6 are expressed on the plasma membrane, where they recognize various bacterial and fungal Pathogen Associated Molecular Patterns (PAMPs) in the extracellular environment [36]. Moreover, surface TLRs, such as TLR2 and TLR4, are also involved in virus detection, as they recognize viral proteins. Interestingly, recognition of viral components by surface TLRs could be seen as viral subversion, since virus activation of TLR2 and TLR4 can promote establishment and perpetuation of viral persistence by inducing IL-10 production [31]. TLRs 3, 7, 8, and 9 are mainly expressed inside cells on endosomal membranes. TLR3 detects double-stranded RNA (dsRNA), a critical intermediate of RNA virus genome replication. TLR7 and TLR8 detect ssRNA produced by viruses, and TLR9 detects unmethylated CpG motifs in bacterial or viral DNA [31,36].

ssRNA and dsRNA are not unique to microbes, but their location in endosomes likely reflects the origin from microbes. Indeed, host cell RNA is not normally present in endosomes, but microbial RNA may end up in endosomes of neutrophils, macrophages, or plasmacytoid dendritic cells (pDCs) when the microbes are phagocytosed by these cells. Enzymatic digestion of the microbes within endosomes will release their nucleic acids so that these are able to bind TLRs in the endosomal membrane. Thus, the endosomal TLRs can distinguish nucleic acids of normal cells from microbial nucleic acids based on the cellular location of these molecules [31,33,36].

TLR3 uses TRIF to activate the inhibitor-kb kinase (IKK) and IKKe/TANK Binding Kinase 1 (TBK1) complexes. TLRs 2, 6, 7, 8 and 9, by contrast, utilize the MyD88 to activate the downstream signaling. In the case of TLR4, downstream signaling requires both MyD88 and TRIF. The major transcription factors activated by TLR signaling pathways are NF-κB, interferon response factor 3 (IRF3), and IRF7. NF-κB essential modulator (NEMO) stimulates the expression of genes encoding many of the molecules required for inflammatory responses, including inflammatory cytokines (e.g., tumor necrosis factor [TNF] and IL-1), chemokines (e.g., CCL2 and CXCL8), and endothelial adhesion molecules (e.g., E-selectin), and costimulatory molecules (CD80, CD86) [38].

**Figure 1:**
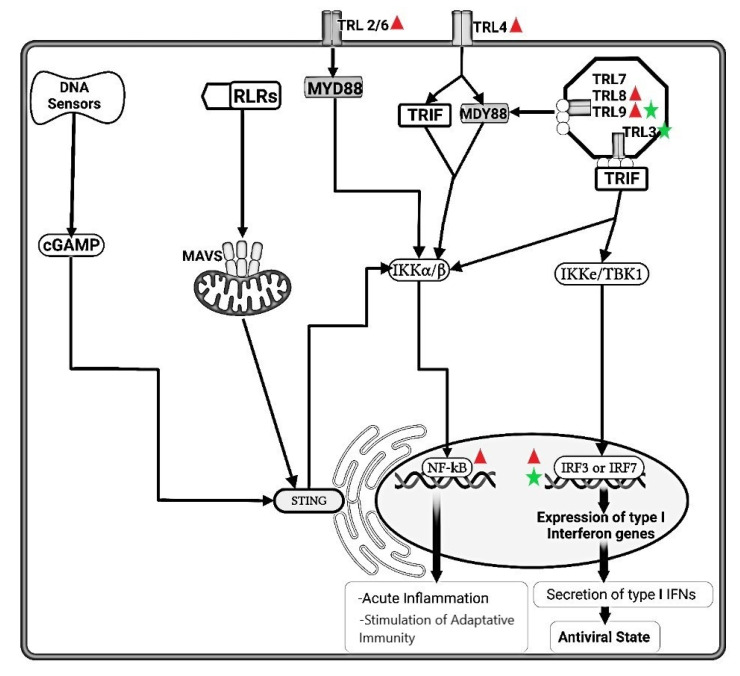
Schematic representation of signaling pathways and functions of Toll-like receptors (TLRs). TLRs 2 and 6 use the adaptor protein MyD88 and activate the transcription factor NF-κB, which induces inflammatory gene expression. TLR3 uses the adaptor protein TRIF, which activates the IRF3 transcription factor and NF-κB. TLR4 uses both MyD88 and TRIF, leading to activation of NF-κB and IRF3 pathways, respectively. TLRs 7, 8, and 9 in the endosome use MyD88, leading to activation of both NF-κB and IRF7, promoting expression of genes whose products mediate inflammation and antiviral defense. DNA viruses are sensed in the cytosol by the presence of the DNA sensors, the cGAS-STING pathway, resulting in the activation of NF-κB and IRFs. RIG-I-like receptors (RLRs), recognize microbial nucleic acids and initiate the MAVs-STING pathways. The pathway targeted by feline coronavirus/feline peritonis virus (FeCoV/FIPV) and feline herpesvirus-1 (FeHV-1) are highlighted using 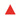 and 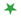, respectively. 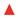 FeCoV infection induced higher gene expression level of TLRs 2, 4, 8 and 9, but not TLRs 3 and 7, suggesting either lack of an appropriate trigger, or virus inhibition of TLR trascripion. The synthesis of accessory proteins 7a and 3 by FeCoV are probably involved in the inhibition of type I IFN synthesis. The FIPV nsp5 produced an inhibition of IRF3 phosphorylation and suppression of type I IFN production. 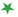 FeHV-1 infection induced an upregulation of TRL9 expression and a downregulation of TLR3. The FeHV-1 US3 protein competitively binds to IRF binding domain hindering dimerization of IRF3. **TIR**, Toll IL-1 receptor; **TLR**, Toll-like receptors; **MyD88**, Myeloid differentiation primary response 88; **TRIF**, TIR-domain-containing adapter-inducing interferon-β; **NF-κB**, Nuclear Factor kappa B; **IRFs**, Interferon Regulatory Factors; **IFN**, Interferon; **IKK**, Inhibitor-KbKinase; **TBK1**, TANK Binding Kinase 1; **cGAMP**, Cyclic guanosine monophosphate-adenosine monophosphate; **STING**, Stimulator of Interferon Genes; **RLRs**, RIG-I-like receptors; **MAVs**, Mitochondrial antiviral-signaling protein; **nsp**, nonstructural protein.

IRF3 and IRF7 promote expression of the genes encoding interferon IFN-α and IFN-β, respectively, which are both type I IFNs that are important for antiviral innate immune responses [36,37] (Figure 1).

Recently, higher gene expression levels of TLRs 2, 4, 8, and 9 have been identified in FIPV and FCoV-infected cats, suggesting their possible role in the pathogenesis of FIP. Furthermore, these molecules were identified as potential target for FIP control [5].

TLR2 in felines is highly expressed in small intestine lamina propria and in mesenteric lymph nodes [37], and its upregulation in FIP could suggest a role as a ligand of the FCoV protein S, since TLR2 has been associated with the detection of the severe acute respiratory syndrome coronavirus (SARS-CoV) S protein in vitro. On the contrary, TLR4, previously associated with protection against murine coronavirus [39], in FIP-positive cats did not reveal a protective effect, although upregulation of TLR4 gene expression in mesenteric lymph nodes was observed [5]. Interestingly, TLR9 gene expression wasnot elevated in the mesenteric lymph nodes of cats with FIP but increased in thoseof FCoV-positive cats without FIP. Indeed, a previous in vitro study highlighted a reduction in viral replication when TLR9 is stimulated with a synthetic CpG ligand before FCoV infection [3]. The increased gene expression in FCoV-infected cats without FIP could indicate that TLR9 helps to prevent disease development, showing a protective effect [5].Given that TLR2 and 4 are typically associated with bacterial infections, there are several hypotheses about the increased levels of gene expression of these TRLs associated with viral infections. TRL4 activity was detected in lung tissues from a mice model infected withseveral respiratory viruses (i.e., SARS-CoV, influenza virus H1N1, and other lung viruses), due to the production of oxidized phospholipids. This molecule, as bacterial LPS, causesthe activation of MyD88 and TRIF, with an overproduction of inflammatory cytokines [37,40]. Moreover, it was suggested that increased levels of gene expression of TRLs 2 and 4 are due to stimulation by co-infecting agents, because of the increased intestinal permeability to microorganisms induced by enteric coronavirus infection and generalized inflammatory state induced by FIPV [5].

Although several studies on SARS-CoV and coronavirus of Middle East respiratory syndrome (MERS-CoV) in humans have demonstrated the role of TLRs 3, 7 and 8 in the evolution of the protective response against coronavirus, the lack of upregulation occurring for TLR3 and TLR7 in FIP-infected cats suggests either the lack of an appropriate trigger, or virus inhibition induced by TLR transcription [5]. SARS-CoV is known to inhibit both TLR3 and TLR7 signaling via papain-like protease activity (PLpro) [5]. This mechanism could also contribute to FCoV infection, although FCoV would be expected to directly affect signaling pathways rather than TLR mRNA levels [5]. In FIP-infected cats, a slightly lower TLR3 gene transcription levelhas been observed in mesenteric lymph nodes showing typical FIP lesions, compared to mesenteric lymph nodes of healthy cats. This could indicate a general systemic stimulus to upregulate TLR3 in FIP, which is locally counteracted by viral inhibition of TLR3. In vitro studies have shown that prior stimulation of TLR3 contributes to the defense against murine coronavirus [41], representing a potential pathway for further researches on FIP [5]. Studies on SARS-CoV and other coronaviruses have demonstrated the association between some genome regions and immunostimulating activity of tumor necrosis factor (TNF)-a, interleukin (IL)-6, and IL-12 produced through TLR7 and 8 [42,43]. Currently, there is no evidence that FCoV, whether or not it is associated with FIP, can adopt the same pathways of immunological stimulation [5].

During HSV infection in humans, TLRs2, 3 and 9 are known to be involved as the host’s first line of defense [25,44,45]; likewise, TLRs 3 and 9 are involved in the FeHV-1 infection [25]. In FeHV-1-infected cats, at 36 h post infection (pi), TRL9 expression is upregulated, while TLR3 expression is downregulated. Interestingly, TLR2 expression levels remain substantially unchanged [25]. FeHV-1-infected Air-Liquid Interface Feline Respiratory Epithelial Cells (ALI-FRECs) mounted both IL-1β and TNFα responses that were significant at 24 pi. Similar observations were made in cats displaying clinical signs of FeHV-1 infection [24,46]. Furthermore, pro-inflammatory cytokines IL-1β and TNFα are known to play an important role in determining the outcome of Herpes simplex 1 (HSV-1) infection [47]. Although further evidence is needed, it is intriguing to consider that during FeHV-1 infection, the increase in IL-1β, TNFα, and IFN-α expression could be attributed to activation of TLR9, rather than of TLR3 [25].

A new formulation of a liposome-TLR complex (LTC) was developed. This includes a TLR9 agonist, a TLR3 agonist, and methylcellulose as a mucosal adhesive agent. The cytokines and cellular immune responses to this LTC were evaluated both in vitro and in vivo. In vitro experiments showed that the LTC rapidly activated cat leukocytes, including upregulation of costimulatory molecules and cytokine production; the in vivo experiments conducted on healthy purpose-bred cats showed that topical administration of the LTC triggers rapid recruitment of monocytes in the nasal and oropharyngeal mucosa [48,49]. Moreover, it has been shown that a single mucosal administration of LTC 24 h before FeHV-1 infection in cats was associated with several positive clinical effects and with decreased shedding of FeHV-1 DNA. Although further investigation is needed, these findings suggest that administration of LTC in cat shelters, which present a higher risk of exposure to FeHV-1 and other pathogens, could significantly improve clinical outcomes, especially in younger subjects [49].

### 2.3. IFNs

Although multiple cytokines and chemokines are produced by different types of host cells during viral infection, IFNs are the main cytokines involved in the antiviral response [50]. Since its first identification in 1957 by Isaac and Lindemann [51,52], it has been determined that there are several types and subtypes of IFNs; these proteins are key elements of antiviral resistance at the cellular level, playing a central role in both innate and adaptive immune responses to viral infections [33,36]. INFs are glycoproteins of 20 to 34 kDa classified into I, II, III types. There are many type I INFs, which are structurally homologous, and include: multiple IFN-α isoforms, a single IFN-β, and other members including IFN-τ, IFN-δ, IFN-ε, IFN-κ, and IFN-ω present in a variable way in different animal species [33,34,36]. Type I IFNs are produced and released by many different cell types within a few hours pi inducing an antiviral state in neighboring cells. Depending on virulence and viral dose, type I IFN can control, or even eliminate, a viral infection before systemic infection or overt disease develops [34]. If the virus overwhelms the early innate immune response, then systemic spread occurs, and disease may be detected clinically [34]. Type II IFN(IFN-γ) regulates both innate and adaptive immunity and defines multiple subtypes of T lymphocytes [33,34]. A third type of IFN, called type III IFN, or IFN-λ, composed of IFN-λ1, -λ2 and -λ3, has also been identified [33,34]. Although these IFNs adapt to different receptors, they share downstream signaling molecules and regulate the same genes [33]. IFN-λ has some peculiar differences compared to IFN-α such as production and action on epithelial cells on mucosal surfaces where it orchestrates both innate and adaptive immune responses to pathogens other than viruses [53].

The most potent stimuli for type I IFNs synthesis are viral nucleic acid. RLRs and DNA sensors in cytosoland TLRs 3, 7, 8, 9 in endosomal vesicles recognize microbial nucleic acids and initiate signaling pathways that activatethe IRF family of transcription factors, which then stimulate the transcription of type I IFNs (Figure 1). The receptor for type I IFNs, a heterodimerof two structurally related polypeptides (IFNAR1 and IFNAR2), binds both IFN-α and IFN-β. This receptor signal activates transcription factors STAT1, STAT2 (JAK-STAT signaling), which finally induce expression of several genes that give cells a resistance to viral infection (antiviral state) [31,33,36].

**Figure 2:**
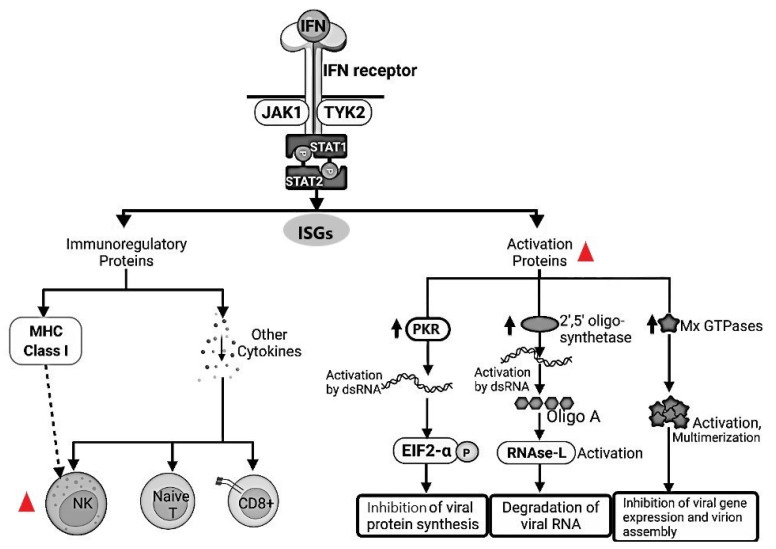
Schematic representation of pathways of type I interferon (IFN) induction and receptor signaling. Type I IFNs bind to receptors (IFNAR) on neighboring uninfected cells and activate JAK-STAT signaling pathways, which induce the expression of genes whose products interfere with viral replication. Type I IFNs also bind to receptors on infected cells and induce expression of genes whose products enhance the cell’s susceptibility to cytotoxic T lymphocyte (CTL)-mediated killing. The pathway targeted by feline coronavirus/feline peritonis virus (FeCoV/FIPV) is highlighted using 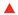. 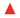 FeCoV infection inhibited the type I IFN synthesis that results in an inhibition of the protein synthesis. Natural Killer (NK) cells are drastically depleted from the peripheral blood mesenteric lymph nodes and spleen in FIPV-infected cats. Moreover, NK showed less cytotoxic activity in FIP-infected cats. **dsRNA**, Double-stranded RNA; **PKR**, Double-stranded RNA-activated protein kinase, **EIF2-a**, Eukaryotic translation initiation factor 2A; **RNAse-L**, Ribonuclease L; **MHC**, Major Histocompatibility Complex; **NK**, Natural Killer; **Naive T**, Naïve T cells; **CD8+**, Cytotoxic T lymphocytes; **JAK1**, Janus Kinase 1; **TYK2**, Tyrosine kinase 2; **STAT1**, Signal Transducer and Activator of Transcription 1; **STAT2**, Signal Transducer and Activator of Transcription 2; **Mx GTPases**, *Mx* dynamin-like *GTPases*; **ISGs**, Interferon stimulated genes.

Type I IFN-induced genes include double-stranded RNA-activated serine/threonine protein kinase (PKR), which blocks viral transcriptional and translational events: 2,5-oligoadenylate synthetase and RNase L, which promote viral RNA degradation; and Mx GTPases proteins that induce inhibition of viral gene expression and virions assembly. Furthermore, type I IFNs cause sequestration of lymphocytes in lymph nodes, thus maximizing the opportunity for encounter with microbial antigens, increase the cytotoxicity of Natural Killer (NK) cells and CD8 and CTLs, promote the differentiation of naive T cells to the Th1 subset of helper T cells, and upregulate expression of class I MHC molecules, and thereby increase the probability that virally infected cells will be recognized and killed by CD8 and CTLs [36,54] (Figure 2).

During co-evolution with their hosts, many viruses have evolved redundant mechanisms to counteract the host immunity for optimal viral adaptation. There is much evidence that coronaviruses have the abilityto evade host IFN response via accessory proteins. These proteins are either involved in the inhibition of IFN synthesis (such as ORF3b, ORF6 and N protein of SARS-CoV), or circumvent the IFN signaling pathway (such as ORF7 protein of transmissible gastroenteritis virus, ORF5a and N proteins of murine herpesvirus and ORF3b, ORF6 and 7a protein of SARS-CoV) [55,56,57]. Porcine epidemic diarrhea virus nonstructural protein 1(nsp1) is known to be the most potent IFN antagonist [55]. Moreover, SARS-CoV PLpro efficiently inhibits activation of the IRF3 pathway by disrupting the interaction between the components of IFN genes stimulator [38,58]. While SARS-CoV nsp14 is an exoribonuclease that can prevent IFN responses by a specific digestion of dsRNA and subsequent removal of RNA-PAMPs [38,59], interestingly, MERSV nsp16 is essential for IFN resistance and viral pathogenesis [38,60].

Despite the still limited information, as well as in other coronaviruses, an accessory protein 7a able to counteract IFN-α-induced antiviral response, has recently been identified in FIPV [19,38,55]. The presence of protein 7a prior to infection does not reduce IFN-α production by FIPV, indicating that protein 7a antagonizes the downstream IFN-producing cascade. Further evaluation of its mechanism showed that protein 7a could interfere with the IFN-α antiviral response only in the presence of one or more proteins encoded by ORF3 (3a or 3b). The synergism of protein 7a and protein 3 (encoded by ORF3) allows for the efficient replication of the FIPV-wild type. Although it is not clear whether this cooperation results from a direct interaction of these proteins, it is possible that they interfere on different IFN-induced pathways that result in the same antiviral effect (e.g., inhibition of protein synthesis) [19,55]. Blocking both IFN-induced pathways could be essential for the virus to overcome the overall negative effect of IFN [55]. Interestingly, deletion ofaccessory ORFs from FIPV wild type does not imply full susceptibility of the virus to IFN, revealing that other viral protein (nsps and/or structural proteins) mayalso participate in the IFN antagonism [55]. More recently, the action of FIPV nsp5 has been demonstrated as a negative regulator that inhibited type I IFN production by cleaving multiple NEMO sites, resulting in the inhibition of IRF3 phosphorylation and in suppression of type I IFN production [38,55] (Figure 1). In addition to the antagonism to type I IFN production, several FIPV strategies of immune evasion, such as retention, internalization, complement block and suppression of lymphocyte proliferation have been described [61,62,63].

As with coronaviruses, during co-evolution with their host, herpesviruses have also evolved a variety of strategies to evade the host’s innate immune response, establishing latent and persistent infections. Multiple anti-IFN response effectors encoded by herpesviruses have been identified. Eleven different proteins have been found in FeHV-1 (including UL30, ICP0, UL11, UL55, UL1, UL45, UL27, UL 3.5, UL48, UL4, and US3) that, cooperating with each other, could contribute to immune evasion of FeHV-1 during infection [64]. US3 of HSV-1 is reported as a multifunctional protein that can regulate viral replication by phosphorylating various viral and cellular substrates. Although the US3 gene is conserved among all alpha-herpesviruses [64], the function of this gene can vary among different species. While the US3 protein of HSV-1 interacts with IRF3 and the kinase activity of US3 phosphorylates IRF3 at Ser175 to inhibit IRF3 dimerization, the US3 protein of FeHV-1 competitively binds to IRF binding domain hindering dimerization of IRF3, regardless of kinase activity [64] (Figure 1).

Since its first detection in humans in 1985, IFN-ωhas been explored as a treatment option for certain diseases and viral infections in humans and other animals [65]. IFN-ω is produced by leukocytes and exhibit antiviral, immunomodulatory, anti-proliferation, and antitumor activities [33,34]. IFN-ω has not yet been discovered in canine and mice species, but it has been identified in felines, horses, pigs, cattle, bats, and humans. IFN-ω ofthese species shares several common characteristics but also some differences. It is antigenically distant from IFN-α and -β and does not react to specific antibodies despite a common mechanism of action [65]. IFN-ω binds to identical receptors and then induce the transcription of MX proteins, ISG, unlike other type IIFNs. IFN-ω shows a moderate degree of cross-species activity and a low level of toxicity that makes it a valid candidate for antiviral pharmacological use in heterologous species. However, there are some limitations to the use in vivo represented by its poor pharmacokinetics and a short half-life [65]. To date, recombinant feline IFN-ω (rfeIFN-ω) is licensed as Virbagen Omega, Virbac, only in Europe, Japan, and Australia for the systemic treatment of canine parvovirus, feline leukemia virus and feline immunodeficient virus infections. Although not licensed for use in other virus infections, it has been shown to be effective against FeHV-1 and FeCoV in vitro and in vivo [66]. The rFeIFN-ω licensed protocol consists of three therapeutic cycles of five daily subcutaneous injections (1 MU/kg), starting on days 0, 14, and 60, respectively [65]. However, its wide-spread use is limited because this protocol is relatively expensive and time-consuming. In addition, alternative protocols, involving subcutaneous and topical administrations, such as oral and intralesional administration, have been developed [65]. No adverse effects have been reported following mucosal administration of rFeIFN-ω to cats, whereas subcutaneous administration may occasionally be associated with mild adverse effects, such as fever, lethargy, vomiting and diarrhea. Topical administration of IFN is feasible for most pet owners and would therefore be a cost-effective treatment option in veterinary clinical practice [65,66,67].

Several studies showed that rFeIFN-ω can be used in beneficial therapies that are impossible in FIP-infected cats. However, these studies were conducted on a limited number (≤12) of cats per group and did not include a control group. Moreover, the effect of rFeIFN-ω has not been reliably confirmed in FIP [68,69]. In a clinical trial, 10^6^ U/kg of rFeIFN-ω was administered daily to 12 FIP-suspected cats until remission, followed by injections every week, and four of the 12 cats lived more than 2 years [68]. In a subsequent case–control study, no significant differences were found between the survival times of cats treated with rFeIFN-ω and those treated with a placebo, although treatment with rFeIFN-ω resulted in significantly lower lymphocyte counts [68].

Interestingly, rFeIFN-ω has been shown to have a dose-dependent inhibitory effect on FeHV-1 replication in vitro [69]. Several studies showed conflicting results about the biological activity of rFeIFN-ω against these two viruses in cats.In a study of 20 cats with FeHV-1-associated ocular keratitis, without a control group, one dose of 0.5 MU/mL rFeIFN-ω was administered five times aday, resulting in significant improvement in ocular signs after 3 weeks of treatment [65]. In subsequent case–control studies, rFeIFN pretreated cats unexpectedly showed no beneficial effects in clinical signs after FeHV-1 experimental infection compared to control group cats despite lower FeHV-1 genome copies [65,70]. Hence, further researches are needed to assess the therapeutic effect of rFeIFN-ω in cats.

### 2.4. Natural Killer Cells

The main host defense strategy against viral pathogens is the elimination of infected cells. This can be achieved by cell-intrinsic mechanisms induced by type-I IFNs and operating in infected cells, or with the help of cytotoxic lymphocytes: NK cells and CD8 T cells [33].

NK cells are specialized, specific cytotoxic lymphocytes lacking an antigen-specific receptor, which can kill not only virus-infected cells but also tumor and stressed cells [71]. NK cells express a large complex of receptors that recognize the expression patterns of their respective ligands on host cells. Receptors on NK cells are both activating and inhibitory, and the function of NK cells is tightly regulated by the balance of activation and inhibitory signals from these receptors. One of the primary receptors on NK cells binds to class I major histocompatibility complex (MHC) proteins and this binding provides a negative (inhibitory) signal for NK cells activation. This allows NK cells to “scan” tissue without harming healthy cells, which are recognized as “self”.

A common evolutionary effect of virus infection is the reduced expression of class I MHC protein on the surface of the infected cell with the aim of avoiding immune responses related to antigen presentation [33,34,71]. The lack of a sufficient MHC ligand to bind the NK cell inhibitory receptor results in activation signals that reach the threshold necessary for cell activation. The receptors that mediate the activation or the inhibition of NK cells to target cell killing are encoded in two large families of genes: the killer immunoglobulin-like receptors (KIR) and the NK receptor complex. NK cells kill virus-infected cells via an identical pathway exerted by antigen-specific cytotoxic T lymphocytes (CTL), inducing apoptosis. This cytocidal activity is pivotal for the control of viral infections because it can eliminate infected cells before producing and releasing the progeny virions. As with CTL, NK cells possess cytosolic granules holding perforin, granzyme A and granzyme B. When these cells are activated, cytosolic granules are oriented toward the target cell and then released. Perforin creates pores in the target cell membrane through which the granzyme proteins enter, and once inside, these proteins induce apoptosis of the target cell through the activation of the pathway of caspases. NK cells also express CD16, a surface receptor for the Fc portion of immunoglobulin G molecules (FcRγIII). This receptor allows NK cells to bind and to lyse antibody-coated target cells through the process of antibody-dependent cellular cytotoxicity. This results in a killing activity identical to the cell-killing mechanism just described but bypassing all the NK cell receptors. Finally, NK cells can also mediate direct killing, being able to efficiently produce and secrete IFN-γ following their activation. IFN-γ secretion by NK cells creates a strong inflammatory environment, activates other cells of the innate and the adaptive immune system, and induces an antiviral state in cells at the site of inflammation [34,71].

The role of NK cells and regulatory T cells (Tregs) in the innate and adaptive cell-mediated immunity, respectively, was investigated in naturally FIPV infected cats [61]. Tregs are a population of CD4^+^ T cells (termed also as CD4^+^CD25^+^Foxp3^+^ for the presence of other two markers) responsible for the immune response regulation mainly due to immunosuppression [11,61]. NK cells and Tregs are drastically depleted from the peripheral blood, mesenteric lymph nodes and spleen in FIP-infected cats, whilst the mesentery and kidneys from cats with FIP show no differences to healthy uninfected control cats. Other regulatory lymphocytes of the CD4^+^CD25^−^Foxp3^+^ and CD3^+^CD8^+^Foxp3^+^ phenotypes were depleted from both blood and lymph nodes. NK cells from lymph nodes of FIP-infected cats also showed less cytotoxic activity than NK cells from the lymph nodes of healthy cats. Therefore, it appears that FIP infection is associated with severe depletion of both NK cells and Tregs, and reduced NK cell function. This could reduce the ability of the innate immune system to attack the virus and to suppress the associated immunologic and inflammatory responses [11,61] (Figure 2).

### 2.5. MicroRNAs

MicroRNAs (miRNAs) are major class of single-stranded noncoding RNAs of approximately 20–24 nucleotides, mainly encoded by non-protein coding regions in the genome, including introns and intergenic regions [72,73,74]. Since the first miRNA identification [75], thousands of miRNAs have been discovered in various plants and animals [72,76]. MiRNAs play an important regulatory role in the innate immune response [72,73], through complete or partial complementary pairing with target genes, resulting in transcriptional degradation or translation suppression [77,78,79]. Several studies have demonstrated that JAK-STAT signaling could be regulated by host miRNAs by targeting key adapter molecules, suppressors of the cytokine signaling (SOCS) family. In general, virus infections can modify the host miRNAs expression profile, indicating that these miRNAs are directly or indirectly involved in the modulation of virus replication [72]. Currently, many miRNAs have been reported to influence virus replication by targeting viral genomes [74,80] and by regulating type I IFNs production or their downstream pathway [81]. During viral infection, host innate immunity is blocked at an early stage. Several studies have suggested that despite the activated type I IFN signaling was rapidly suppressed following FeHV-1 infection [64], some miRNAs were still upregulated to enhance IFN signaling pathways [82,83]. Interestingly, some microRNAs have also been reported to inhibit virus replication by directly targeting viral genomes [74,80]. Given the critical roles of miRNAs in regulating type I IFN signaling, it is unknown whether the host cell uses these miRNAs to restart the IFN signaling pathways during FeHV-1 infection.To explore the vital role of miRNAs involved in the host resistance process to FeHV-1 infection, high-throughput sequencing of small RNAs after FeHV-1 infection was performed, showing that miR-26a was significantly upregulated at the time of infection [73]. A more recent study demonstrated that miR-26a plays an important role in host defense against FeHV-1 infection; in fact, miR-26a can suppress FeHV-1 replication, increasing STAT1 phosphorylation and promoting type I IFN signaling cascades by directly targeting SOCS5.

Further action is the negative regulation of the STAT signaling pathway [73]. A subsequent study showed that miR-101 is also involved in the regulation of innate immunity during FeHV-1 infection enhancing type I IFN antiviral signal and increasing IFN production to inhibit FeHV-1 replication [72].

Further investigation is necessary to evaluate the role of different miRNAs in innate immunity during viral infection caused by both herpesviruses and other viral families.

## 3. Conclusions

In recent decades, there has been tremendous progress in the characterization of the innate immune recognition pathways exploited by viral pathogens. Although some obvious gaps remain, the role of TLRs, IFNs, NK cells, and miRNAs in antiviral defense is now better understood and recognized. Most IFN-induced antiviral gene products have yet to be functionally characterized. These proteins likely interfere with multiple steps in viral infection cycles. Their functional redundancy hinders the recognition of their contributing role in antiviral defense. Other outstanding questions include the elucidation of the mechanisms that control the expression of ligands for activation and inhibition of NK receptors. FIPV and FeHV-1 represent examples of how tight the links are between innate and other immunity mechanisms such as antibody-dependent enhancement (ADE) of virus infection and the pathogenetic activities of viruses. Ultimately, the greatest challenge will be the application of accumulated knowledge to the management—and potentially even eradication—of major viral infections that continually threaten both animals and humans.

## Data Availability

Not applicable.
